# Editorial: Recent innovations in breast reconstructive surgery: a continuous debate

**DOI:** 10.3389/fsurg.2025.1632595

**Published:** 2026-01-09

**Authors:** Andrea Lisa

**Affiliations:** 1 PhD Program in Applied Medical Surgical Sciences, Department of Surgical Sciences, University of Rome “Tor Vergata”, Rome, Italy; 2 Humanitas University Department of Biomedical Sciences, Humanitas University, Milan, Italy

**Keywords:** breast cancer, breast implant, breast reconstruction, fat graft, microvascular flap

Breast reconstruction remains a cornerstone of multidisciplinary breast cancer management, continuously evolving through the integration of new technologies, refined surgical techniques, and a stronger emphasis on patient-reported outcomes. The studies featured in this special issue reflect the dynamic nature of this field, covering the full spectrum from oncologic safety and aesthetic restoration to quality-of-life and complication prevention. Together, they illustrate how innovation must coexist with critical evaluation—each step forward generating new questions and perspectives.

In a rigorous Bayesian network meta-analysis, Li et al. evaluated the *efficacy and safety of various flaps in autologous breast reconstruction* across ten clinical studies involving 871 patients. By comparing six commonly used flap techniques, the authors provided a comprehensive hierarchy of outcomes ranging from flap loss to psychosocial well-being. Their analysis showed that while the TUG flap minimized total flap loss, the Four-flap technique achieved superior satisfaction and sexual well-being scores. These findings underscore the importance of tailoring reconstruction choices not only to anatomical suitability but also to expected functional and psychological outcomes—a key step toward patient-specific reconstructive planning supported by quantitative evidence.

Precision in oncologic safety continues to be central to breast surgery. Li et al. developed an *intraoperative nomogram* capable of predicting secondary margin positivity in breast-conserving surgery (BCS) when using frozen section analysis. Their model, based on 348 patients, demonstrated strong predictive performance with AUC values up to 0.83 and reliable calibration across validation sets. By combining pathology and clinical data available intraoperatively, the proposed tool supports real-time surgical decision-making—potentially reducing re-excisions, anesthesia time, and hospital costs. This study exemplifies how machine-learning principles can translate into pragmatic tools enhancing oncologic accuracy without compromising operative flow.

The psychometric dimension of reconstruction outcomes was explored by Daneshi et al., who conducted a *bibliometric analysis* on the top 100 cited papers addressing quality of life in implant-based breast reconstruction (IBR). Analyzing more than 64,000 patients, they found that the vast majority of studies were of moderate evidence level (OCEBM II–III) and that validated PROMs—particularly the BREAST-Q—were inconsistently used. The lack of level I evidence in this field highlights an urgent need for robust prospective designs integrating standardized PROMs. Their work calls for the reconstruction community to place patient-reported metrics on equal footing with clinical endpoints, aligning research output with what truly matters to patients: comfort, confidence, and body image restoration.

In a rare randomized controlled trial, Xie et al. investigated how wearing an external breast prosthesis influences body posture after unilateral mastectomy. Among 240 participants, the weighted prosthesis significantly improved forward head posture, shoulder asymmetry, and scapular or neck tilts compared with cotton substitutes. These results emphasize that external devices can contribute not only to body image but also to biomechanical stability—bridging rehabilitation and aesthetics. Such studies reinforce the idea that reconstruction extends beyond surgery, encompassing the broader physical and psychosocial reintegration of breast cancer survivors.

Han et al. presented a novel vertical muscle-sparing latissimus dorsi flap for the treatment of *chronic radiation-induced chest wall ulcers*. Their case series demonstrated that this approach reduces operative time and morbidity while maintaining reliable perfusion and functional recovery. By avoiding patient repositioning and preserving muscle integrity, this modification simplifies complex reconstructions often performed in high-risk, irradiated fields. The technique exemplifies how revisiting classic flaps through a minimalist lens can achieve both surgical efficiency and tissue preservation—two priorities in modern reconstructive philosophy.

Lisa et al. reported a single-center preliminary study assessing the *efficacy of axillary dead-space closure* (quilting) after mastectomy with lymph-node dissection and immediate prosthetic reconstruction. Among 81 patients, quilting significantly reduced drain duration (16 vs. 20 days) and clinically significant seromas (3.7 vs. 11%). It also lowered implant removal rates. Mechanical dead-space obliteration, though technically simple, proved a decisive factor for postoperative safety and cost reduction. The findings advocate for routine incorporation of axillary quilting in reconstructive protocols, representing an elegant low-tech solution to a persistent clinical problem.

Complication prevention also extends to microbiological vigilance. Lisa et al. conducted a multicentric retrospective study on *periprosthetic infections following implant-based reconstruction*, analyzing 214 infected cases out of nearly 10,000 reconstructions. Gram-positive bacteria, particularly *Staphylococcus aureus* and *S. epidermidis*, dominated the isolates, though Gram-negative multidrug-resistant strains appeared more frequently in overweight and smoking patients. The correlation between host factors and pathogen distribution provides new insights for tailored prophylaxis and empiric antibiotic strategies. This large dataset bridges plastic surgery with infectious-disease medicine, underscoring that reconstructive success increasingly depends on interdisciplinary collaboration.

Across these contributions, several unifying themes emerge. First, data-driven personalization is reshaping reconstructive decision-making—from Bayesian meta-analysis of flap outcomes to nomogram-guided oncologic margins. Second, functional and psychological outcomes are gaining parity with traditional metrics of survival and symmetry. Third, innovation does not always mean complexity: sometimes, technical refinements such as flap orientation or quilting sutures achieve the most meaningful clinical impact. Finally, the interplay between aesthetics, biomechanics, and infection control exemplifies the multidisciplinary essence of modern breast reconstruction.

As editors, we are witnessing a paradigm shift in which reconstructive surgery transcends its restorative mandate to become a fully integrated, evidence-based component of cancer care. The studies presented here collectively push the boundaries of safety, precision, and patient-centered design—while acknowledging that continuous debate remains essential to progress. In breast reconstruction, innovation is not an endpoint but a conversation—one sustained by critical analysis, shared data, and above all, the lived experience of our patients.

